# Cross-species immunoprotective antigens (subolesin, ferritin 2 and P0) provide protection against *Rhipicephalus sanguineus* sensu lato

**DOI:** 10.1186/s13071-023-06079-3

**Published:** 2024-01-03

**Authors:** Ismail Zeb, Luís Fernando Parizi, Muhammad Israr, Itabajara da Silva Vaz, Abid Ali

**Affiliations:** 1 Department of Zoology, Abdul Wali Khan University Mardan, Mardan, 23200 Pakistan; 2 Centro de Biotecnologia and Faculdade de Veterinária, Universidade Federal do Rio Grande do Sul, Campus do Vale, Porto Alegre, RS 91501-970 Brazil; 3 Pakistan Science Foundation, Islamabad, Pakistan

**Keywords:** Anti-tick vaccine, Cross-protection, Cocktail, *Rhipicephalus sanguineus* sensu lato

## Abstract

**Background:**

Tick control is mostly hampered by the rise of acaricide-resistant tick populations. Significant efforts have focused on developing alternative control methods, including cross-species protective and/or cocktail-based anti-tick vaccines, to achieve protection against various tick species.

**Methods:**

In this study, full-length open reading frames encoding subolesin (SUB) from *Rhipicephalus microplus* and ferritin 2 (FER2) from *Hyalomma anatolicum* as well as the partial 60S acidic ribosomal protein (P0) from *R. microplus* were cloned, expressed in *Escherichia coli* and used as vaccine antigens against *Rhipicephalus sanguineus* sensu lato (*R. sanguineus* s.l.) infestation in rabbits.

**Results:**

In silico analyses revealed that the SUB, P0 and FER2 proteins were antigenic and displayed limited similarity to the host's homologous proteins. The proteins shared identities of 97.5%, 100% and 89.5% with their SUB, P0 and FER2 *R. sanguineus* s.l. orthologous sequences, respectively. Antibodies against each recombinant protein cross-recognized the native proteins in the different tissues and developmental stages of *R. sanguineus* s.l. Overall efficacy of the SUB, FER2 and cocktail (SUB+FER2+P0) vaccines against *R. sanguineus* s.l. infestation was 86.3%, 95.9% and 90.9%, respectively.

**Conclusions:**

Both mono-antigen and the cocktail anti-tick vaccines affected the biological parameters of *R. sanguineus* s.l. infestation in the rabbit model, which could be extrapolated to its infested host under natural conditions. These findings support the possibility of using mono-antigenic and cocktail-based vaccines for large-scale anti-tick vaccine development against multiple tick species.

**Graphical Abstract:**

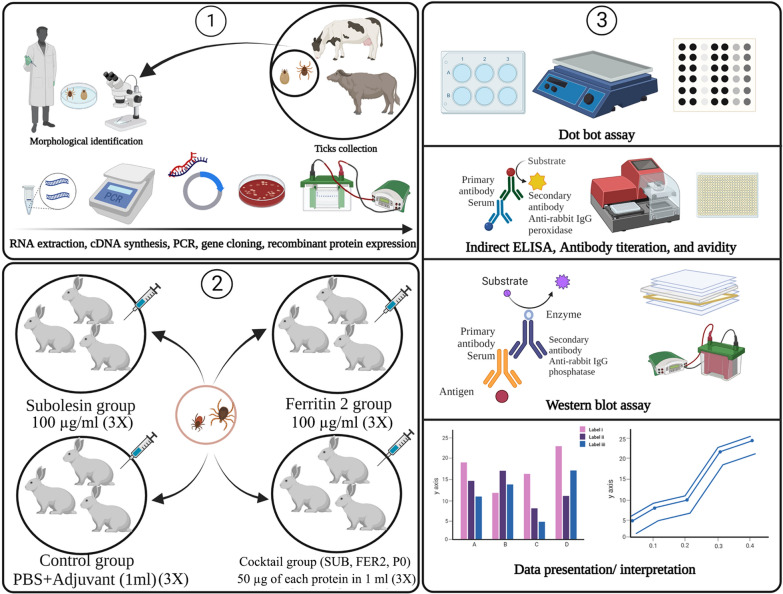

**Supplementary Information:**

The online version contains supplementary material available at 10.1186/s13071-023-06079-3.

## Background

The cattle tick *Rhipicephalus microplus*-derived midgut protein Bm86 is currently the only commercialized anti-tick vaccine antigen available [1–3], but its anti-tick vaccine efficacy varies against different strains and species of ticks from different parts of the world [4]. This limitation has led several research groups to test novel antigens for anti-tick vaccine development [5], but only a few antigens have been found to induce adequate immune protection against multiple tick species [6–13], and some antigens failed to affect the physiological parameters of the tick tested[14–16]. Consequently, to date, various anti-tick vaccines are still in the pipeline of development and have not progressed to commercialization [17, 18]. Moreover, there is a growing consensus that anti-tick vaccines which are cross-protective and/or cocktail-based are essential for enhancement of vaccine efficacy against a range of tick species [10, 19, 20]. For example, the proteins Bm86, glutathione S-transferase (GST), ATAQ, cement protein/truncated recombinant 64P proteins (64TRPs), subolesin (SUB), ferritin 2 (FER2) and 60S acidic ribosomal protein (P0) have been shown to provide varied protection against multiple tick species [13]. However, only a few studies have evaluated the efficacy of cocktail-based anti-tick vaccines [2, 8, 19, 21, 22], and more often, the protection mechanisms are poorly understood [17]. To date, several potential tick-derived proteins have shown strong cross-reactivity, indicating that a combination of multiple antigens may achieve a cumulative anti-tick vaccine efficacy against tick infestation [9]. It has been suggested that such anti-tick vaccines can be improved to induce a broader immune protection with the identification of proteins that share conserved sequences among various tick species [10, 23] or which are expressed in different tick developmental stages [3]. Additionally, some of the tick protective antigens in the cocktail can provide long-lasting immunity to prevent or reduce tick infestations and pathogen transmission in several hosts [17].

Over the past decades, a growing number of tick-derived proteins have been evaluated against *Rhipicephalus* species [24]. Among these, *Rhipicephalus sanguineus* sensu lato (*R. sanguineus* s.l.) has been used as a tick model in various vaccine experiments with rabbits as host [25]. Although some of these tick proteins have been shown to induce some degree of protection, none of them alone showed sufficient efficacy for the development of a commercial vaccine [26]. SUB, FER2 and P0 have been specifically identified as candidate immunoprotective antigens [13]. SUB is a transcription factor that is active in multiple cellular activities, including blood-feeding, reproduction, development and the innate immune response [27]. The primary function of the gut iron-binding FER2 protein is the transportation of iron molecules [7], while the major function of the P0 protein is the assemblage of the 60S ribosomal subunit [28].

Based on the essential roles of these regulatory proteins in tick physiology, their immune-protective potential has been assessed, revealing varied protection against tick species in different hosts [13]. Moreover, the use of these antigens in cross-protective and/or cocktail-based anti-tick vaccine trials against *R. sanguineus* s.l. infestation could facilitate the development of improved anti-tick vaccines [29]. The aim of the present study was to analyze the *R. microplus*-derived SUB and *Hyalomma anatolicum*-derived FER2 as mono-antigenic vaccines, as well as *R. microplus*-derived P0 protein in combination with SUB and FER2 as a cocktail vaccine (SUB+FER2+P0) to protect rabbits against *R. sanguineus* s.l. infestation.

## Methods

### Ethics statement

The vaccination experiment was conducted at the Faculdade de Veterinária, Universidade Federal do Rio Grande do Sul (UFRGS), Porto Alegre, Brazil. The experiment was approved and carried out according to the guidelines of the Ethics Committee on Animal Experimentation of UFRGS (no. 37568) and by the Advanced Studies and Research Board Committee of Abdul Wali Khan University Mardan (Mardin, Pakistan) under number Dir/A&R/AWKUM/2021/5466.

### Collection and identification of ticks

Following written and oral consent obtained from cattle owners, cattle were examined for the presence *R. microplus* and *H. anatolicum* ticks at various locations in the arid zone of Mardan (72.0791°E, 34.1617°N, Khyber Pakhtunkhwa Province (KP), Pakistan. The collected ticks were washed immediately in phosphate buffer saline (PBS) for 1 min and then air-dried [30]. All collected ticks were morphologically identified at the species level using published dichotomous keys [31, 32] under a stereo zoom microscope (model SZ61; Olympus Corp., Tokyo, Japan).

### *Rhipicephalus sanguineus* s.l. ticks and rabbits for vaccination

Parasite-free *R. sanguineus* s.l. belonging to the tropical lineage was collected in Uberlândia, Brazil (− 48.27538°E, − 18.91460°N) and maintained by experimental infestation on rabbits (*Oryctolagus cuniculus*) at the Faculdade de Veterinária, Porto Alegre, Brazil. Ticks that dropped off the host were maintained at 28 °C and 85% relative humidity (RH) for oviposition. The vaccination trial was performed on 4-month-old New Zealand rabbits weighing approximately 2 kg. The rabbits were kept in isolated cages during the experiment.

### Nucleic acid (DNA, RNA) extraction and cDNA synthesis

Partially engorged females of *R. microplus* and *H. anatolicum* were individually dissected in ice-cold PBS (pH 7.2). Whole tick-derived tissues were homogenized in a single 1.5-ml tube and subjected to DNA extraction using a DNA extraction kit (Qiagen Ltd., West Sussex, UK), and RNA extraction using TRIzol Reagent (Ambion^®^, Life Technologies, Thermo Fisher Scientific, Waltham, MA, USA) following the manufacturer’s instructions. The quantity and purity of extracted DNA and RNA were assessed using a Nano-Drop spectrophotometer (Nano-Q; OPTIZEN, Daejeon, South Korea).

A 1-µg sample of DNase-treated RNA was incubated at 70 °C for 5 min with 1 µl of 100 µM oligo (dT)^18^ and 10 µl DEPC-treated water (Thermo Fisher Scientific). The reaction was snap-chilled on ice for 1 min, followed by the addition of 4 µl first-strand reaction buffer (5×), 20 U/µl RiboLock RNase inhibitor, 2 µl of dNTPs (10 mM) and 200 U/µl RevertAid M-MuLV RT (Thermo Fisher Scientific). The reaction was incubated at 42 °C for 1 h followed by termination at 70 °C for 5 min. The complementary DNA (cDNA) concentrations and purity were determined using a Nano-Drop spectrophotometer (Nano-Q; OPTIZEN).

### Primer synthesis and PCR amplification

Tick-borne pathogens were screened using sets of primers, including the 18S ribosomal RNA (rRNA) gene of piroplasms (*Theileria*/*Babesia* spp.), *Rickettsial gltA* and *Anaplasma* spp. 16S rRNA gene as previously described (Table 1). In each PCR reaction, PCR water was used as a negative control while *Theileria annulata*, *Rickettsia massiliae* and *Anaplasma marginale* DNA were used as the positive control for the screening of tick-borne pathogens. To amplify the full-length open reading frame (ORF) sequences of SUB and P0 from *R. microplus* and those of FER2 from *H. anatolicum*, primers were designed based on the retrieved homologous sequences from the GenBank for SUB (KM115651, EU301808, JQ922399, JX431507-09, JQ713774-77, JQ713779-80, JQ713782-83, JQ713785), FER2 (KT924235-47) and P0 (KC845304, KP087926). Degenerated primers were used in SUB and P0 cloning (Table 1).

**Table 1:** Primers for tick-borne pathogens screening, and amplification of tick open reading frame coding genes

Organism	Primer sequence	Amplicon (bp)	References
*Piroplasms (Theileria/Babesia* spp.)/18S rRNA gene	F: ACCGTGCTAATTGTAGGGCTAATAC R: GAACCCAAAGACTTTGATTTCTCTC	897	[33]
*Rickettsial gltA*	F: GCAAGTATCGGTGAGGATGTAAT R:CTTCCTTAAAATTCAATAAATCAGGAT	401	[34]
*Anaplasma* spp./16S rRNA gene	F: GGTACCYACAGAAGAAGTCC R: TGCACTCATCGTTTACAG	345	[35]
*Tick’s ORF coding genes*			
Subolesin	F: ATGGCTTGYGCRACATTAAAGCG R: TTACGACAAATAGCTGGGCG	486	This study
P0	F1: ATGGTCAGGGAGGATAAGAC F2: ATTGTGAACGGCCTGAAAAACCTGA R: YYTAGTCGAAGAGTCCGAAGCCCAT	957	This study
		234	This study
Ferritin 2	F: ATGGGCAACAACCTGAACGAACAG R: TTAGGTACGCAGCTGCTGATCCAG	531	This study
Actin	F: TCAGGTCATCACCATCGGCAAC R: GTACATGGTGGTGCCGCCG	184	[36]

Degenerated nucleotides are underlined

*F* Forward,* R* reverse,* PO* 60S acidic ribosomal protein,* rRNA* ribosomal RNA

PCR and thermocycling conditions for screening the tick-borne pathogens were as previously described [33–35]. For the amplification of full-length ORF sequences, a total reaction volume of 25 µl was prepared containing a template cDNA (500 ng/μl), 1× PCR buffer, 0.2 mM dNTPs, 3 mM MgCl2, 1 U Taq DNA polymerase, nuclease-free water (Thermo Fisher Scientific) and 0.5 mM each forward and reverse primers. The thermocycling conditions were: an initial denaturation at 94 °C for 4 min; followed by 35 cycles of denaturation at 94 °C for 30 s, annealing at 60 °C (SUB and FER2) and 50 °C (P0) for 30 s and extension at 72 °C for 60 s; with a final extension at 72 °C for 10 min. A similar PCR assay was prepared, and a targeted partial 234-bp sequence of the amplified P0 gene was further amplified using a new forward primer with the previously used reverse primer (Table 1). A negative control without cDNA and a positive control containing cDNA and a specific set of actin primers were prepared to check the integrity of cDNA [36]. The PCR assays were performed in a PCR thermocycler (model T100; Bio-Rad Laboratories Inc., Hercules, CA, USA), and the obtained PCR products were analyzed in a 1% agarose gel stained with ethidium bromide.

### Purification and cloning of PCR products

The amplified SUB, FER2 and P0 PCR products were purified using the GENECLEAN II kit following the manufacturer’s instructions (MP Biomedicals, Solon, OH, USA) and individually ligated into a pGEM-T vector (Promega, Madison, WI, USA) following the manufacturer’s instructions. The recombinant plasmids were transformed into the *Escherichia coli* Top10 host strain (GenScript, Piscataway, NJ, USA) using the heat shock method [37] and subsequently dispersed on Luria Bertani (LB) agar plates supplemented with ampicillin (50 µg/ml). The plates were kept overnight at 37 °C. A single colony was picked and grown in 25 ml of LB broth (with 100 µg/ml ampicillin), and the recombinant plasmids were recovered using a mini-prep protocol [37]. The sequences were confirmed by introducing a new set of primers comprising *Nde*I and *Hin*dIII restriction sites and the N-terminal 6×His-tag DNA sequence for subsequent cloning into an expression vector. Recombinant plasmids confirmed by PCR were digested with *Nde*I and *Hin*dIII restriction enzymes and sequenced. The resultant products were individually ligated into the pET-30a(+) vector (GenScript), as per the manufacturer’s instructions. Each recombinant pET-30a(+)/SUB (rSUB), pET-30a(+)/FER2 (rFER2) and pET-30a(+)/P0 (rP0) were transformed into the *E. coli* BL21 Star™ (DE3) expression system (GenScript) using the heat shock method, and the recombinant products were recovered using a miniprep protocol [37]. The ligation was confirmed by PCR screening, digestion with restriction enzymes and DNA sequencing.

### In silico analyses

BLAST analysis was performed using nucleotide sequences to identify the corresponding conserved homologous nucleotide and protein sequences in hard ticks [38]. Also, alignment and identity matrix studies for *R. microplus*-derived SUB and P0 protein sequences with *R. sanguineus* s.l. SUB (JX193845) and P0 (XM_037651068) and for *H. anatolicum*-derived FER2 protein sequence with *R. sanguineus* s.l. FER2 (XM_037648827) were conducted in BioEdit software version 7.2.5 [39, 40]. Antigenic peptides in SUB, FER2 and P0 proteins were predicted at the 0.5 threshold value using online ElliPro: Antibody Epitope Prediction tools provided in the IDEB analysis resources website (http://tools.immuneepitope.org) [41]. Antigenic index plots were determined in the Jameson–Wolf algorithm [42] using Lasergene software version 7.0.0 (DNASTAR, Madison, WI, USA). In silico-based predicted molecular weights for SUB, FER2 and P0 proteins were determined using the Compute pI/Mw tool in Expasy [43].

### Expression and purification of recombinant proteins

The recombinant *E. coli* BL21 Star™ (DE3) host strains were individually cultured on LB agar plates supplemented with 100 µg/ml ampicillin for 16 h at 37 °C. Individual colonies were picked and subcultured separately in 25 ml LB broth (with 100 µg/ml ampicillin) overnight at 37 °C. The culture broths were centrifuged for 10 min at 16,000* g* at 4 °C and the pellets re-suspended in 500 ml of fresh LB broth and then incubated at 37 °C until optical density (OD_600_) reached 0.4. Expression was induced with 1 mM of Isopropyl b-d-1-thiogalactopyranoside (IPTG) for 5 h at 37 °C and monitored by sodium dodecyl sulfate-polyacrylamide gel electrophoresis (SDS-PAGE) in a 12% separating gel. Induced cells were harvested by centrifugation at 16,000* g* for 10 min at 4 °C, and the obtained pellets were washed twice in PBS (pH 7.2). Pellets for each of the rSUB, rFER2 and rP0 proteins were separately re-suspended in lysis buffer (50 mM Tris, 150 mM NaCl, 0.1% Triton X-100, pH 8.0), incubated for 1 h at 22 °C and lysed using a Vibra-Cell VCX 500–700 ultrasonic homogenizer (Sonics Inc., San Jose, CA, USA) at 5 cycles of 30 pulses for 30 s. The lysate was centrifuged at 16000* g* for 10 min at 4 °C, following which the supernatant was filtered through a 0.45-µm porosity filter (MilliporeSigma, Burlington, MA, USA); the targeted rSUB, rFER2 and rP0 proteins were then purified by nickel affinity column chromatography (GenScript). Purification was performed in PBS, pH 7.3 (washing and binding buffer) and 10 mM Tris HCl (pH 8.0), 0.5 M NaCl and 20 mM imidazole (elution buffer). Fractions containing the eluted proteins were dialyzed in PBS (pH 7.2) for 12 h at 4 °C. Protein purity was verified by SDS-PAGE in a 16% separating gel under reduced conditions. The concentrations were determined according to the standard bovine serum albumin (BSA) using the Bradford assay [44]. The purified recombinant proteins were stored in a storage buffer (50 mM NaHCO_3_, 150 mM NaCl, 10% glycerol, pH 10.0) at − 70 °C for further use.

### SDS-PAGE and western blot assay

The rSUB, rFER2, and rP0 proteins were verified by SDS-PAGE in a 16% gel under a reduced condition and quantified using a Nano-Drop spectrometer (Nano-Q; OPTIZEN). For Western blot [45], 5 µg of protein sample was mixed with 15 µl loading buffer (5% SDS, 5% Tris [pH 6.8], 0.2% bromophenol blue and 20% glycerol) and heated at 100 °C for 10 min. Each protein sample (20 µl), protein marker (5 µl; Bio-Rad Laboratories) and BSA standard (5 µl) were gel-electrophoresed at 100 V for 20 min in stacking gel and 150 V for 1 h in running gel, at 4 °C. The protein samples were then transferred to a nitrocellulose membrane in 12 mM carbonate buffer (pH 9.9) at 70 V for 1 h at 4 °C. The membranes were blocked with blocking buffer (1× PBS containing 5% skim milk) and incubated with shaking at room temperature (RT) for 1 h, followed by 3 washes with the same buffer. The membranes were then treated with 1:1000 dilution of rabbit-anti-His monoclonal antibodies (GenScript). Detection was performed in alkaline phosphatase buffer (100 mM Tris–HCl, 100 mM NaCl, 5 mM MgCl2) with the addition of 0.3 mg/ml nitro blue tetrazolium (NBT; Thermo Fisher Scientific) and 0.15 mg/ml 5-bromo-4-chloro-3-indolyl phosphate (BCIP; Thermo Fisher Scientific) [16].

### Vaccination trial

**Fig. 1:**
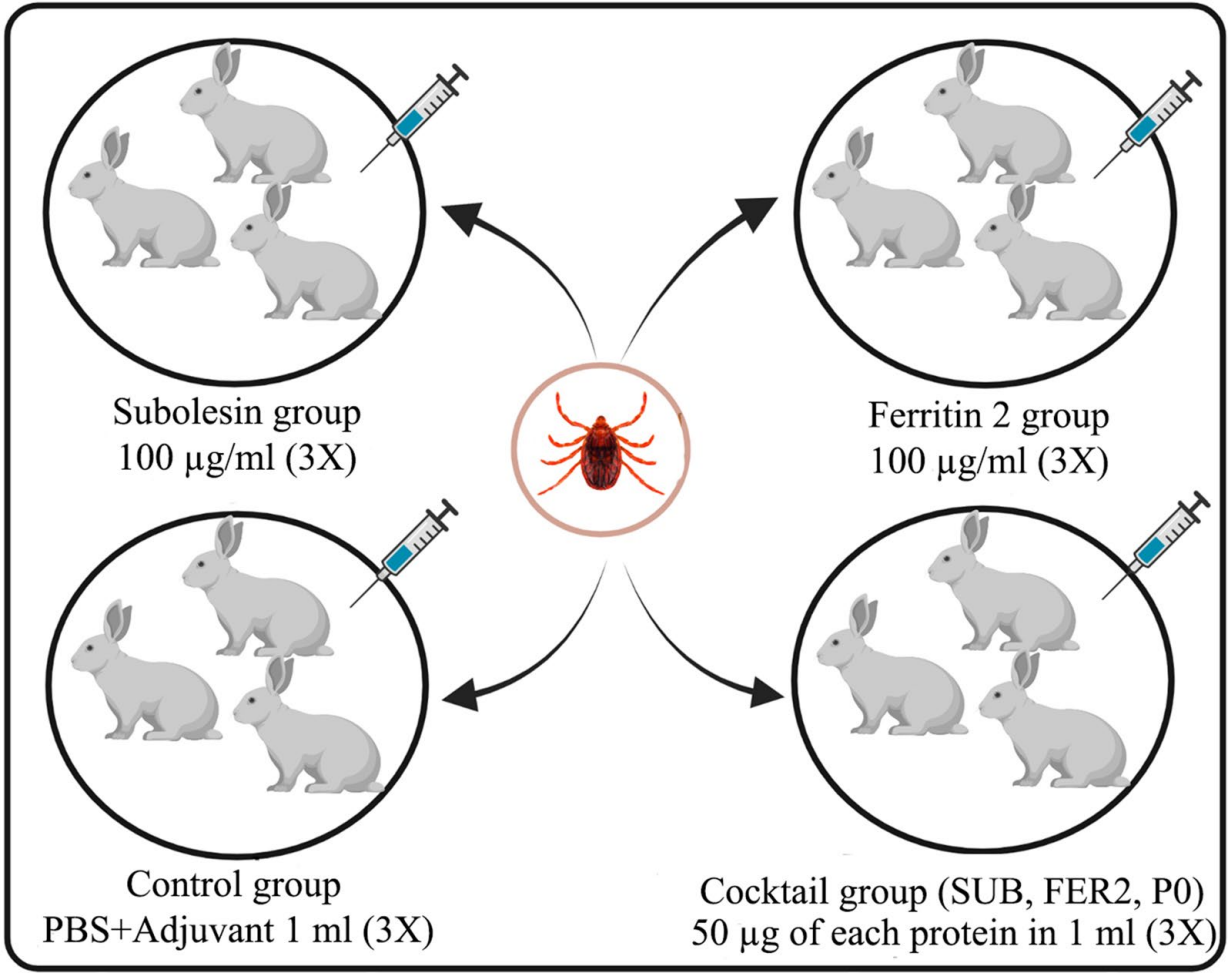
Overview of rabbit immunization strategy with tick-derived proteins. FER2, Ferritin 2 protein from *Hyalomma anatolicum*; PBS, phosphate-buffered saline; PO, partial 60S acidic ribosomal protein from *Rhipicephalus microplus*; SUB, subolesin protein from *R. microplus*

Twelve rabbits divided into four groups (3 vaccinated groups and 1 control group) of three rabbits each were kept in individual cages for the vaccination trial. The rabbits were subcutaneously inoculated with 100 µg of rSUB (SUB group), 100 µg of rFER2 (FER2 group), 50 µg of each of the rSUB, rFER2 and rP0 proteins (cocktail group) or PBS alone (control group) emulsified with the Montanide adjuvant (Montanide™ ISA 61 VG; Seppic, La Garenne-Colombes, France). The rabbits were immunized a total of 3 times at 14-day intervals. For *R. sanguineus* s.l. infestation, each rabbit was infested with 40 nymphs and 12 adults (6 males and 6 females) placed in separate chambers on the back of the ears (Fig. 1). Elizabethan collars were bound around the rabbit’s neck to avoid chamber removal. The ear bags were checked on a daily basis for dropped nymphs and females. Engorged nymphs until molting and engorged females until oviposition were maintained in individual 1.5-ml tubes at 28 °C and 85% RH [16].

Rabbit blood samples were collected before each inoculation and at 2 weeks after the third dose and 30 days post-infestation (day 75). Sera were obtained by centrifugation at 5000* g* for 10 min and stored at − 20 ℃ for further analysis.

### Indirect enzyme-linked immunosorbent assay, serum titration, and avidity index

Ninety-six-well plates (Sigma-Aldrich, St. Louis, MO, USA) were independently coated with 0.1 μg/50 µl of each rSUB, rFER2 or rP0 protein in coating buffer (0.05 M carbonate-bicarbonate, pH 9.6) and incubated at 4 °C overnight. The wells were then blocked with 0.05% Tween-20 (Plus One Tween-20; Pharma Biotech) in 1× PBS (PBST) at 37 °C for 1 h, followed by the addition of 100 µl of rabbit specific anti-sera in 1:500 dilution in PBST and incubation at 37 °C for 2 h. Anti-rabbit IgG peroxidase conjugate (Sigma Chemical Co., St Louis, MO, USA) was diluted (1:5000) in PBST, and loaded onto the wells of each plate at 100 µl/well, followed by incubation at 37 °C for 1 h. After each incubation step, the plates were washed 3 times with PBST. The reaction was performed using 100 µl of *O*-phenylenediamine dihydrochloride (Thermo Fisher Scientific) in citrate buffer (pH 5.0), and the plates were incubated for 15 min in the dark at RT. The reaction was stopped with the addition of 50 µl H_2_SO_4_ (12.5%). The OD values were measured at 492 nm using a microplate spectrophotometer (Spectramax Microplate Reader; Molecular Devices LLC, San Jose, CA, USA). The results of the cocktail vaccine were independently evaluated for each antigen. Antibody levels were considered to be positive when the average serum OD readings were twofold higher than the average OD reading of the corresponding pre-immune serum [16].

For serum titration, primarily seven serial dilutions of antibodies, ranging from 1:128,000 to 1:8,192,000 were tested. Pre-immune, post third inoculation and post-infestation sera against each rSUB, rFER2 and rP0 protein were tested. A similar indirect enzyme-linked immunosorbent assay (ELISA) protocol was used as described above.

The serum avidity index (AI) was also evaluated in the pre-immune and post first and third immunization sera. A slight modification was introduced into the indirect ELISA protocol. Briefly, the plates were washed with denaturant buffer (100 µl/well) containing 0 M, 4 M or 6 M urea in PBS/0.05% Tween-20, pH 7.2 for 3 min at RT after the addition of the primary antibodies (1:500 dilution). The serum AI was calculated as the OD ratio of bound to unbound antibodies with or without urea treatment [46]. The analysis was performed using average values of experiments carried out in triplicate.

### Tick tissues cross-reactivity

A cross-recognition assay of hyperimmune sera for rSUB, rFER2 and rP0 was performed for their respective native proteins in the *R. sanguineus* s.l. tissues. The native proteins were extracted from engorged female tissues (gut, salivary glands and ovary) at developmental stages (egg, larva and nymph), following a previously described protocol [16]. Total native proteins (10 µg) were individually resolved by SDS-PAGE in a 16% separating gel and transferred to nitrocellulose membranes as described in section SDS-PAGE and western blot assay. The membranes were first blocked, then incubated with immunized/control rabbit sera (1:100 dilution) for 2 h, followed by three washes with blocking buffer and finally by incubation with 1:5000 diluted anti-rabbit IgG phosphatase conjugate on shaker at RT for 1 h. After this step, a similar protocol was followed as mentioned in section SDS-PAGE and western blot assay.

### Statistical analysis

The overall efficacy of cross-protective and cocktail anti-tick vaccines was calculated considering only statistically significant differences in the number of nymphs and molting nymphs, number of recovered engorged females, egg weight and number of hatched larvae between ticks fed on vaccinated rabbits and those fed on control rabbits. The individual protection parameters were calculated with Student's t-test using Microsoft Excel version 10.0.19045. The overall protection was calculated using a previously described formula by taking into account the number of recovered engorged female ticks [47]. The obtained AI results were compared by the Chi-squared t-test, considering *P*-value < 0.05 to indicate a significant difference, using GraphPad Prism software, Window version 5.0 (GraphPad Software, San Diego, CA, USA).

## Results

### Sequence identities

The full-length ORF sequences of *R. microplus* SUB and P0, and of *H. anatolicum* FER2 were 486, 957 and 531 bp, respectively, and the predicted amino acid sequences of SUB, PO and FER2 were 161, 318 and 176, respectively. Nucleotide and deduced protein sequence identities between *R. microplus* and *R. sanguineus* s.l. for SUB were 97.52% and 97.5%, respectively; for P0, these were 94.87% and 98.75%, respectively. Similarly, the nucleotide and deduced protein sequence identities between *H. anatolicum* and *R. sanguineus* s.l. FER2 were 87.5% and 89.5%, respectively. The *R. microplus* P0 protein was 70% identical to the mammalian host *Bos taurus* P0 protein (AAX09097). Therefore, a 234-bp partial nucleotide sequence corresponding to 78 immunogenic amino acids of the C-terminal region of the P0 gene was amplified to produce a partial sequence of P0 protein; this partial P0 sequence was 100% identical between *R. microplus* and *R*. *sanguineus* s.l. The *R. microplus* and *H. anatolicum* ticks were negative for any targeted pathogens (data not shown); thus, the cDNA derived from the pathogen-free ticks was used in the sequence characterizations. The full-length ORFs encoding SUB, FER2 and P0 were deposited into Genbank under accession numbers ON886329, OP219721 and ON921298, respectively.

### In silico analysis

**Fig. 2:**
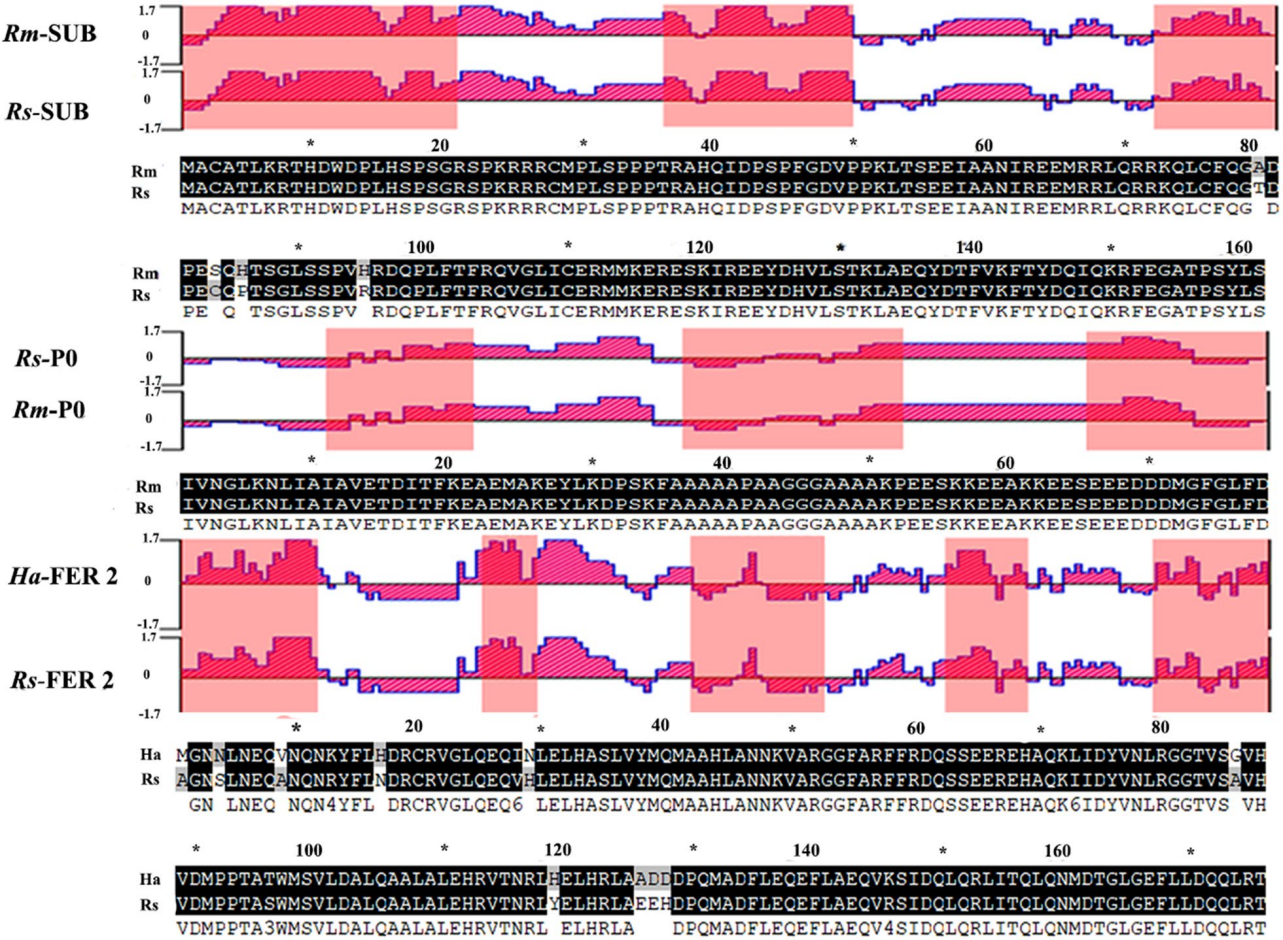
Comparative analysis of conserved and antigenic regions for SUB and partial P0 between *Rhipicephalus microplus* (*Rm*) and *Rhipicephalus sanguineus* sensu lato (*Rs*), and for FER2 between *Hyalomma anatolicum* (*Ha*) and *R. sanguineus* sensu lato protein sequences. Jameson–Wolf algorithm predicted the antigenic index plots showing regions with high antigenicity and identity. The pink, gray and black colors in the aligned sequences represent B-cell epitopes, non-conserved amino acids and 100% sequence identity, respectively. FER2, Ferritin 2 protein; PO, partial 60S acidic ribosomal protein; SUB, subolesin protein

Potential linear B-cell epitopes were predicted for each of the three proteins (Table 2). Comparative analysis of the SUB, partial P0 and FER2 protein sequences with those of *R. sanguineus* s.l. orthologs showed high sequence identity in several antigenic regions (Fig. 2).

**Table 2:** Potential linear B-cell epitopes in subolesin, partial 60S acidic ribosomal protein and ferritin 2 protein sequences

Protein	Potential linear B-cell epitopes
SUB	1-MACATLKRTHDWDPLHSPSGRSPKRRRCMPLSPPPTRAHQI-41, 72-RKQLCFQGADPESQHTSGLSSPVHRDQP-99, 145-YDQIQKRFEGATPSYLS-161
P0	11-IAVETDITFKE-21, 37-AAAAPAAGGGAAAAKP-52, 66-EEEDDDMGFGLFD-78
FER2	1-MGNNLNEQVNQNKYFLHDR-19, 43-AHLANNKVARG-53, 78-NLRGG TVSGVHVDMPPTATWMS-99, 120-ELHRLAADDDPQ-131, 156-TQLQNMD TGLGEF LLD QQLRT-176

*FER2* Ferritin 2 protein from *Hyalomma anatolicum*,* PO*, partial 60S acidic ribosomal protein from *Rhipicephalus microplus*,* SUB* subolesin protein from *R. microplus*

### Expression and purification

The molecular weights of the rSUB, rFER2 and partial rP0 proteins, including histidine tags, as determined from their SDS-PAGE mobility, were approximately 20, 21 and 10 kDa, respectively (Additional file 1: Figure S1). These sizes are compatible with the in silico predicted molecular weights for rSUB (19.5 kDa), rFER2 (20.9 kDa) and rP0 (9.07 kDa).

### Immunogenicity analysis

**Fig. 3:**
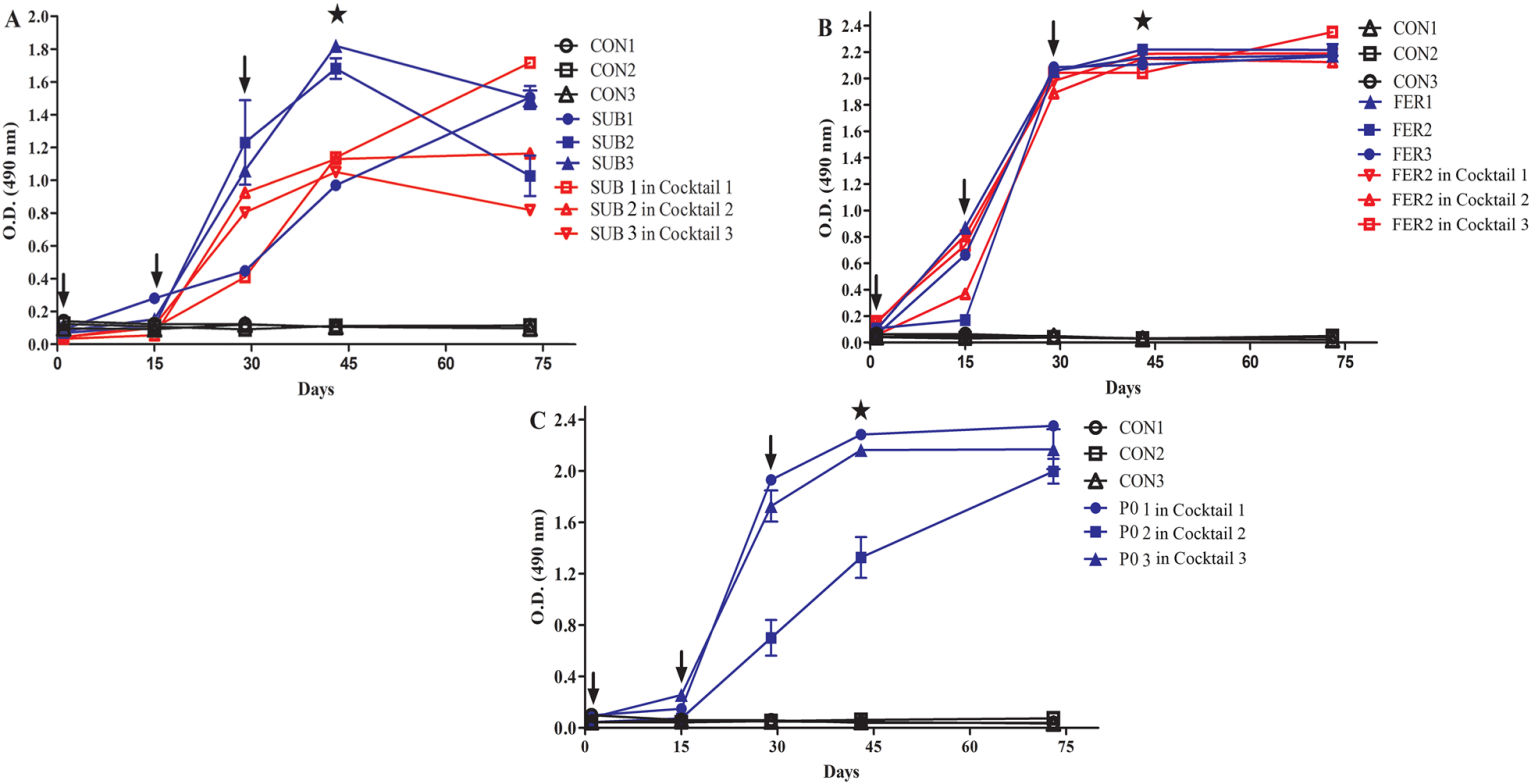
Detection of humoral responses by ELISA in rabbits of control group (CON1, CON2, CON3) and experimental groups immunized with recombinant Subolesin (Rabbits; SUB1, SUB2, SUB3) and the cocktail (**a**), recombinant Ferritin2 (Rabbits; FER1, FER2, FER3) and the cocktail (**b**) and recombinant P0 protein (Rabbits; P0 1, P0 2, P0 3) in the cocktail (**c**). Arrows and stars indicate the immunization and infestation days, respectively. CON, Control; ELISA, enzyme-linked immunosorbent assay; FER2, ferritin 2 protein; O.D., optical density; PO, partial 60S acidic ribosomal protein; SUB, subolesin protein

The ELISA showed the kinetics of the humoral response of the immunized rabbits against rSUB, rFER2 and each rSUB, rFER2 and rP0 proteins in the cocktail (Fig. 3a–c). After two inoculations, the rabbit’s sera recognized their respective inoculated proteins, whereas sera from the control group did not show reactivity. Immunized rabbits showed stable antibody levels after the third inoculation up to the post-infestation period. Two weeks after the third inoculation, at the tick infestation stage, the highest sera antibody titer in the mono-antigenic rSUB- and rFER2-immunized rabbits was 4,096,000 and 8,192,000 (Additional file 2: Table S1), respectively. The highest serum antibody titer of the rabbits immunized with the cocktail vaccine for rSUB, rFER2 and rP0 was 2,048,000, 8,192,000 and 4,096,000 (Additional file 2: Table S1), respectively. The antibody levels showed that all of the tested proteins were immunogenic in rabbits.

**Fig. 4:**
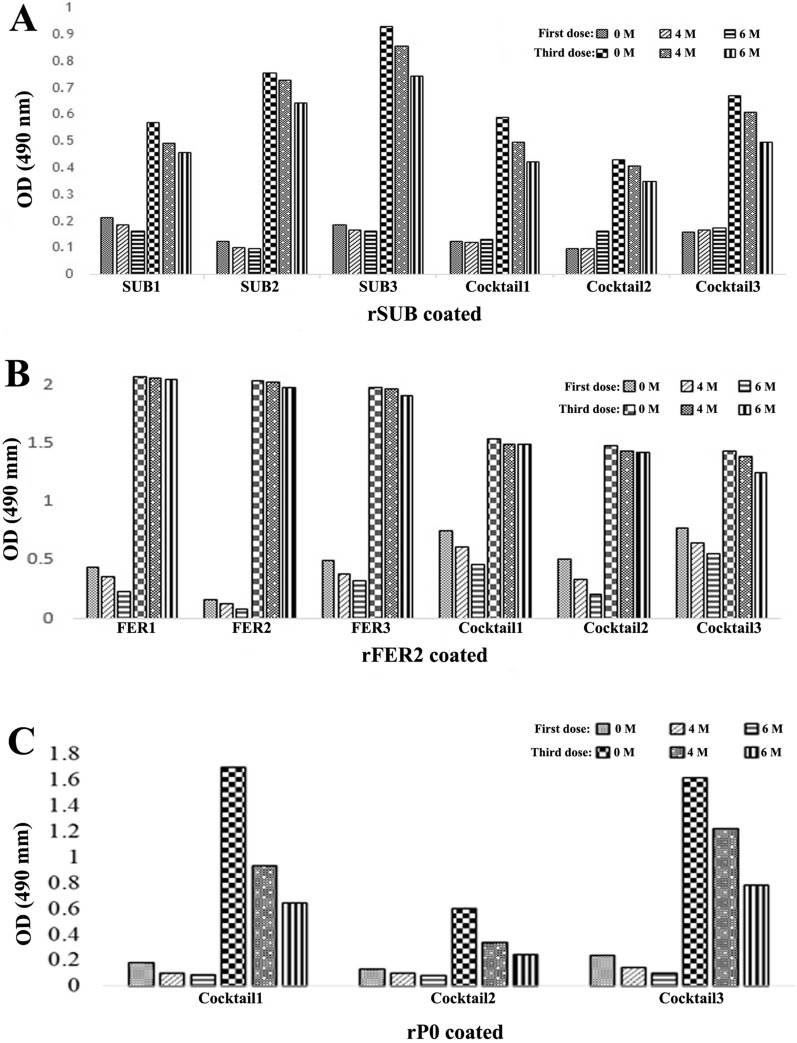
Avidity index of rabbit sera immunized with tick proteins. First and third inoculation antibodies (1:500) against rSUB (**a**), rFER2 (**b**) and rP0 (**c**) proteins. FER2, Ferritin 2 protein; OD, optical density; PO, partial 60S acidic ribosomal protein; rFER2, recombinant ferritin; rPO, recombinant PO; rSUB, recombinant subolesin; SUB, subolesin protein. Statistical analysis was performed by Chi-squared t-test (p < 0.05)

The serum AI for rSUB and rFER2 was 1.1 (Fig. 4a) and 1.6 (Fig. 4b), respectively. Similarly, the AI of the cocktail sera for rSUB, rFER2 and rP0 was 0.9 (Fig. 4a), 1.5 (Fig. 4b) and 1.2 (Fig. 4c), respectively. Taken together, the results showed that the avidity was increased during the immunization program and that there was a significant difference (*P* < 0.05) in the AI among the sera obtained after the first and third doses in all three inoculations.

**Fig. 5:**
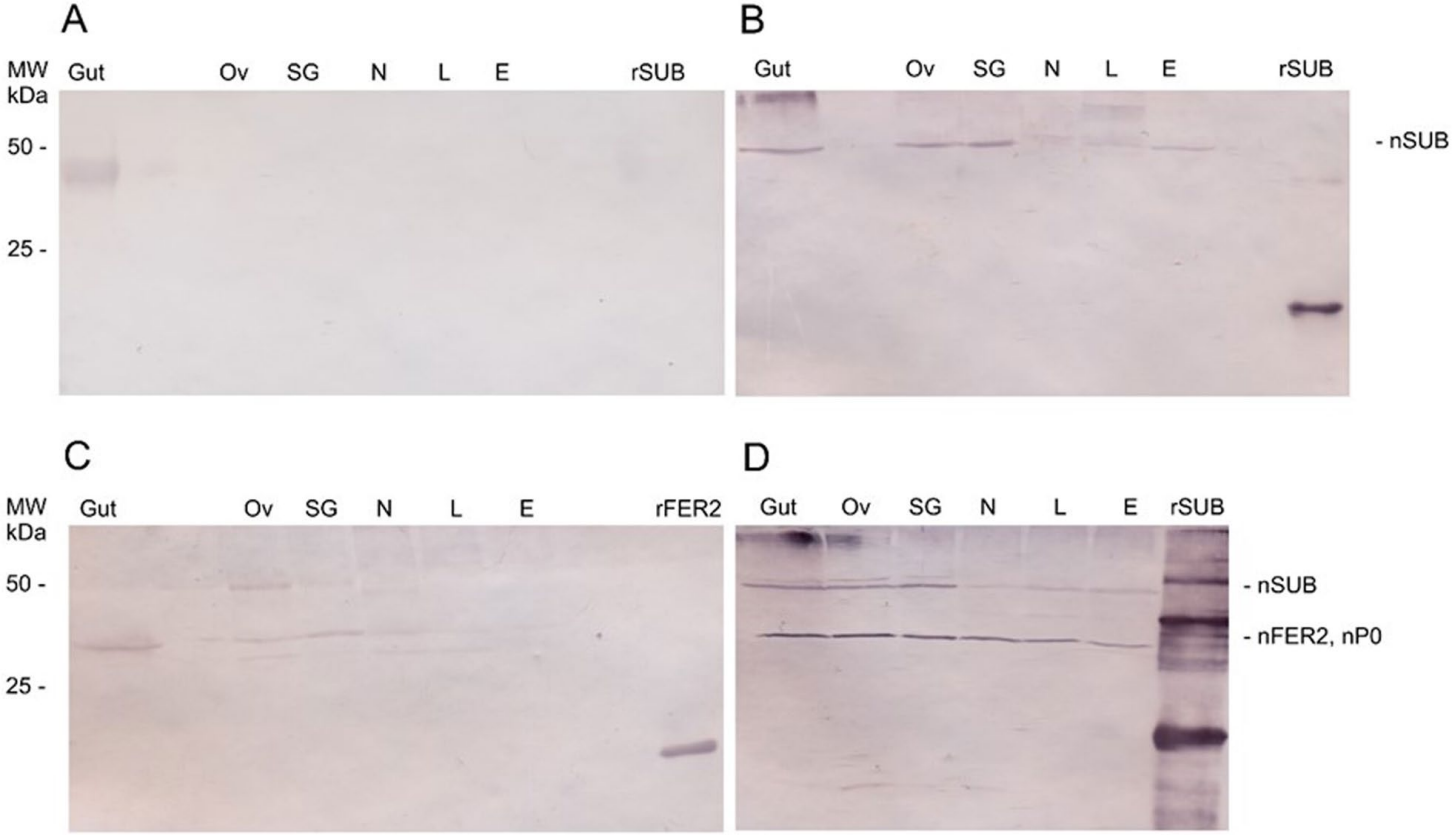
Sera cross-recognitions to native *Rhipicephalus sanguineus* sensu lato SUB, FER2 and P0. By western blot, *R. sanguineus* sensu lato tissue extracts were analyzed using rabbit sera (1:100) from the control group (**a**), anti-subolesin group (**b**), anti-ferritin 2 group (**c**) and anti-cocktail group (**d**). Alkaline phosphatase reactions were performed with nitro blue tetrazolium (NBT) and 5-bromo-4-chloro-3-indolyl phosphate (BCIP). Anti-IgG alkaline phosphatase rabbit sera conjugate (1:5000) was used as secondary anti-body. E, Egg; L, larvae; MW, molecular weight; N, nymph; nFER2, native ferritin 2; nP0, native P0; nSUB, native subolesin; Ov, ovary; SG, salivary glands; rFER2, recombinant ferritin; rSUB, recombinant subolesin.

Sera antibodies against recombinant proteins cross-recognized the native SUB (approx. 50 kDa), FER2 (approx. 30 kDa) and P0 (approx. 30 kDa) proteins from *R. sanguineus* s.l. tissues (gut, ovary and salivary glands), nymphs, larvae and eggs (Fig. 5b–d). The apparent molecular mass was around 50 kDa for SUB and 30 kDa for FER2, since SUB can form dimers [49] and FER2 possesses glycosylation sites [50]. A non-specific band was observed on gut tissue; however, no cross-recognition was observed in the remaining respective tissues and developmental stages against control sera (Fig. 5a).

### Vaccine efficacy against *R. sanguineus* s.l. infestation in rabbits

The feeding capability of nymphs and adult ticks in the control group was greater than that in the immunized groups (Table 3). The number of nymphs, weight of engorged nymphs and molting capability of nymphs were lower in all three immunized groups compared to the control group (*P* < 0.05). There was a decrease of 52.5%, 64% and 51% in the number of viable nymphs for the rSUB, rFER2 and cocktail vaccines, respectively. The weight of engorged ticks, and oviposition and larvae hatching rate were significantly lower in ticks fed on immunized rabbits than in those fed on control rabbits (*P* < 0.05), with the exception of the number of recovered engorged female ticks and the SUB group hatching rate (*P* > 0.05). The overall efficacy for rSUB, rFER2 and cocktail vaccines against *R. sanguineus* s.l. infestation was 86.3%, 95.9% and 90.9% based on adult female numbers, respectively (Table 3).

**Table 3:** Biological parameters of *Rhipicephalus sanguineus* sensu lato ticks fed on immunized rabbits and control rabbits

Groups	Nymphs				
	Engorged^a^	Weight^b^	Molting^c^		
Control	31 ± 3.4	3.75 ± 0.13	26 ± 3		
rSUB	19.3 ± 2.08 (37.6%*)	2.92 ± 0.05 (22%*)	12.33 ± 1.52 (52.5%*)		
rFER2	16 ± 2.6 (48%*)	2.93 ± 0.08 (21.7%*)	9.33 ± 3.75 (64%*)		
Cocktail	22.3 ± 2.08 (27%*)	3.11 ± 0.19 (17%*)	12.66 ± 1.54 (51%*)		

	Adult females				
	Engorged^a^	Weight^b^	Egg laying^d^	Egg fertility^e^	Total vaccine efficacy^f^
Control	5.33 ± 1.15	162.9 ± 13.1	0.56 ± 0.004	0.21 ± 0.035	
rSUB	3 ± 0 (43.75%)	127.8 ± 5.17 (21.5%*)	0.26 ± 0.05 (53%*)	0.138 ± 0.089 (34.3%)	86.3%^g^
rFER2	3 ± 0 (43.75%)	124.1 ± 12.5 (23.8%*)	0.25 ± 0.03 (55%*)	0.104 ± 0.031 (50.4%*)	95.9%^h^
Cocktail	3.33 ± 0.5 (37.5%)	107.8 ± 23.3 (33.8%*)	0.32 ± 0.12 (43%*)	0.095 ± 0.018 (54.8%*)	90.9%^h^

Values in table are presented as the mean ± standard deviation, with the reduction in percentage given in parentheses

* rFER2* Recombinant ferritin2;* rPO* recombinant partial 60S acidic ribosomal protein,* rSUB* recombinant subolesin

*Significant difference from control at *P-*value < 0.05 was performed using Student's t-test

^a^Mean values for engorged nymphs and engorged female ticks fed on rabbits

^b^Mean values for weight (mg) of engorged nymphs and engorged female ticks

^c^Molting capability

^d^Whole egg weight per total female’s weight

^e^Whole larvae weight per total egg weight

^f^Vaccine efficacy = 100 × [1 − (RN × VN × RA × OA × FE)], where RN, VN, RA, OA, and FE are mean values of the number of nymphs, molted nymphs, number of engorged females, egg weight and hatched larvae in the immunized group relative to that in the control group, respectively

^g^Since the RA and FE were not significantly different in this group, these values were considered to be 0% of protection in the total vaccine efficacy formula

^h^Since the RA was not significantly different in this group, this value was considered to be 0% of protection in the total vaccine efficacy formula

## Discussion

Vaccination with tick-derived antigens provides varied protection against tick species of the same genus and/or of different genera [13, 18, 48]. This variation may be due to antigenic differences among the tick-derived proteins that reduce the protection against challenges with other tick species [13]. However, the reasons for these variations in protection have not yet been fully elucidated. Despite these gaps in knowledge, efforts have led to the evaluation of cross-protective and cocktail-based anti-tick vaccine approaches that could enhance the immune protection against different tick species in various geographical regions of the world [9, 29]. In the present study, recombinant SUB, P0 and FER2 proteins from *R. microplus* or *H. anatolicum* were used as vaccine antigens against *R. sanguineus* s.l. infestation in rabbits. Immunization with the selected tick immune-protective proteins was based on their conserved sequences and importance in tick physiology [28, 49, 51]. It is important to note that the adult phase is the major stage involved in pathogen transmission and that the tick-pathogen interaction greatly influences the expression of tick genes and transcriptional shifts [35]. Therefore, ticks that were PCR-negative for infestation were selected for the experiments.

The ELISA revealed that all of the inoculated proteins were immunogenic and induced a humoral immune response in all vaccinated groups, which persisted until the end of experiment. Similar results using these proteins as antigens were obtained in previous studies [7, 28, 49, 52]. It is assumed that antigen-specific antibodies in immunized hosts enter into the body of ticks through the blood meal, bind to the targeted organs and then disrupt vital functions, leading to the death of the ticks [7, 48, 53]. In the present study, a difference in antibody titration was observed between rabbit sera from the FER2 group, with this protein showing a higher antibody titration than the SUB and P0 proteins. The authors of a previous study reported similar results, observing a non-significant difference between anti-pP0 titers and titers of the pP0–Bm86 conjugate [54]. The decrease in antibody titration may be related to the half amounts of antigens present in the cocktail vaccine. This result implies that the ticks may have ingested a higher amount of anti-FER2 antibodies during blood-feeding [48]. The antibody titration in the rabbits vaccinated with the cocktail was high due to the large amounts of antibodies against each antigen. Similarly, in a previous study, the host receiving a cocktail of antigens showed a higher antibody titer than those receiving a single antigen [19]. Differences in protein metabolism between tick species and immunologic interference or antigenic competition with other antigens in combination may affect the anti-tick vaccine efficacy. However, our observations in the present study suggested a limited competition between the antigens in the cocktail proteins; thus, it is possible that cocktail vaccination could result in high protection and synergistic immunity against several tick species. For example, Willadsen et al. [19] reported that the immune protection was enhanced against Bm91 but it did not impair the immune responses of its own cocktail constituent Bm86. In contrast, the cocktail of *Haemaphysalis longicornis*-derived GST (rGST-Hl) and *R. microplus*-derived *Boophilus* Yolk pro-cathepsin (rBYC) and vitellin-degrading cysteine endopeptidase (VTDCE) showed a lower production of antibodies against rBYC and rVTDCE in comparison to the antibodies against rGST-Hl [8]. Indeed, the cocktail used in the present study was shown to be immunogenic since the cocktail serum recognized the constituting antigens. Herein, the mono-antigenic and cocktail immunizations induced a significant increase in the antibody avidities between the first and third inoculations. Higher avidity of antibodies is desirable for a vaccination strategy that seems to play a critical role in the development of protective responses. Moreover, high-avidity antibodies against tick-derived proteins could enhance the bonds to their specific target, consequently interfering with their biological activities [55]. Therefore, investigating antibody avidity is necessary to obtain an adequate immune response, evaluate vaccination efficacy and develop vaccines [56, 57].

Antibodies against rabbits vaccinated with rSUB, rP0 and rFER2 reacted with native proteins in different tissues (gut, ovary and salivary glands) and developmental stages (larvae, nymphs and eggs) of *R. sanguineus* s.l. Similarly, antibodies detected native SUB in the tick midgut and orthologous akirins (AKR) [2, 58], and native FER2 in hemolymph, mid-gut and salivary glands [59]. Induced antibodies against partial rP0 protein in the cocktail recognized the native P0 protein in different tissues and developmental stages which was compatible with the total molecular size of the native P0 protein that was detected at approximately 35 kDa in *R. microplus* [52], and at approximately 34 kDa in *Ornithodoros erraticus* and *Ornithodoros moubata* [60]. Moreover, SUB and FER2 expression in various tissues and developmental stages have been demonstrated for several other ticks [7, 49, 51]. The molecular mass of native proteins was larger than that expected, which was due to the formation of common dimers in SUB/AKR with functional implications [49] and glycosylation in FER2 [50]. Based on the amino acid sequences, the molecular mass of native midgut SUB in the *R. haemaphysaloides* and that of FER2 in *O. erraticus* and *O. moubata *were slightly larger than expected based on previous studies; however, antibodies are specific to their respective epitopes [50, 58]. A faint detection of control sera with the tick’s gut tissue was observed, possibly due to a secondary antibody reactivity against the host rabbit’s sera that specifically binds to the tick gut tissue, since the ticks were fed on rabbits [48]. The adjuvant suitability and dose of the antigens can be determined by high antibody titer [61]. Several adjuvants, including Montanide, that have been exploited in anti-tick vaccine formulations have shown potency and minimal induction of side effects [17]. In the present study, we used the Montanide ISA 61 VG water-in-oil emulsion as adjuvant for vaccine formulation; this emulsion is stable and robust, and induces strong and long-lasting protection for multiple antigens [50, 61]. There is a consensus that antigen dose is directly related to the humoral immune responses and vaccine protection efficacy [62]. Indeed, using the same concentration of antigen in the cocktail as used in the mono-antigenic vaccines may trigger undesired immune responses, including inter-molecular competition [17]. In the present study, vaccinated hosts received three doses and the optimum concentration of vaccine, in accordance with commonly practiced protocols reported previously [7, 10, 49, 63], which were determined on the basis of antibody titer.

The SUB, FER2 and cocktail vaccine significantly reduced the number of nymphal and adult ticks of *R. sanguineus* s.l., showing 86.3%, 95.9% and 90.9% overall efficacy, respectively. The overall efficacy of the vaccines resulted from a reduction in both the recovered number of nymphs and the molting capability of the nymphs; reduced oviposition of engorged adult females of the SUB, FER2 and cocktail groups; and reduced larval hatching rate in the FER2 and cocktail groups. The number of engorged females used in the vaccination experiments were non-significant between vaccinated and control groups, as observed in previous studies [48, 63]. The results of the present study support those of previous experiments in showing the protective effects of these proteins when used separately and in different combinations against different tick species [2, 7, 10, 28, 49, 51]. For example, anti-tick vaccine efficacy for rSUB against tick infestations with homologous and heterologous tick strains ranged between 37.4% and 97% [3, 49, 63, 64], and for rFER2, the anti-tick vaccine efficiency ranged between 49 and 98% [7, 48, 51], depending on differences in the experimental conditions, such as tick species, developmental stages, hosts and/or adjuvant composition [64].

A number of cocktail anti-tick vaccines have shown a significant increase in the protection against tick infestation compared to mono-antigenic vaccines [2, 8, 17, 21, 22, 55, 65], suggesting the possibility of combining multiple tick-derived antigens to effectively control tick infestations [2, 8, 66]. For example, a cocktail of rGSTs from *Rhipicephalus appendiculatus, Rhipicephalus decoloratus, R. microplus, Amblyomma variegatum* and* H. longicornis* showed a significant humoral immune response and reduced the number of *R. sanguineus* s.l. ticks on rabbits by 35% [55]. Similarly, a cocktail of rGSTHI, rBYC and rVTDCE showed higher antibody production and induced 61.6% protection against *R. microplus* infestation on cattle [8]. The SUB protein together with its ortholog akirin in a cocktail vaccine enhanced the anti-tick efficacy of the vaccine against* Ixodes ricinus* and *Dermacentor reticulatus* on rabbits [12]. In another study, SUB conjugation to the P0 peptide resulted in 86.6% anti-tick vaccine efficacy against adult *H. longicornis* as compared to 79.3% by SUB alone [65]. The cocktail of *R. appendiculatus-*derived rRAS-1 and rRAS-2 serpins and a combination of rRAS-3 and rRAS-4 with *R. appendiculatus-*derived 36-kDa immuno-dominant protein in a cocktail highly reduced an *R. appendiculatus* infestation on cattle [22, 66]. Similarly, vaccination with Bm91 enhanced the immune protection against *R. sanguineus* s.l. infestation but did not impair the immune responses against Bm86 [19]. Notably, the final protection conferred by the cocktail vaccine in the present study significantly enhanced protective responses, either similar to or even surpassing those induced by a cocktail of proteins against multiple tick species [2, 8, 22, 55]. Specifically, our cocktail exhibited a higher efficacy than mono-antigenic GST, 64TRP, Bm86, Aquaporin and ATAQ, falling within the range of P0 immunizations against *R. sanguineus* s.l. infestation [11, 16, 23, 26, 28, 67, 68]. Cocktail vaccines have many potential advantages, such as having the potential to induce an immune response against a targeted tick species, or even a cross-protective immune response against several tick species [17, 55]. While antagonistic and synergistic effects of antigens in cocktail vaccines have been reported, few studies have comprehensively evaluated the performance of cocktail antigens against ticks [8].

While a slight decrease was observed in the efficacy of the cocktail vaccine as compared to FER2 protein alone, this decrease did not affect the overall efficacy of the former. Similarly, the authors of a previous study also observed a slight decrease in efficacy when using P0 protein in combination with Bm86 protein against *R. sanguineus* s.l. infestation in dogs [54]. Also, the highest anti-tick vaccine efficacy for *A. variegatum*-, *R. decoloratus*- and *R. appendiculatus-*derived SUB against *R. appendiculatus* was 90%, 85% and 89%, respectively; this was similar to the anti-tick vaccine efficacy obtained by a cocktail of all three SUB antigens (92%) [49]. In contrast, the cocktail of *I. scapularis*-derived 4D8, 4F8 and 4E6 was not as effective as 4D8 alone in terms of reducing tick infestations [21]. In another study, FER2 protein was reported to have shown variable efficacy against several tick species, but it reached its highest efficacy against *I. ricinus*, encompassing even Bm86, while the minimum efficacy was observed against *R. microplus* (64%) [7]. It has been suggested that the use of FER2 alone can reduce tick infestation against certain tick species but that protection against multiple ticks may require the use of FER2 in combination with other antigens to enhance the efficacy of the anti-tick vaccine. Since, anti-tick cocktail vaccines potentially cost more to produce than mono-antigenic vaccines, further studies are necessary to evaluate the economic values of cocktail-based anti-tick vaccines. As compared to one-host ticks, the control of two- or three-host ticks would require more expansive control approaches in order to successfully reduce the different life stages of the target ticks [62]. Therefore, alternative strategies, including chimeric-based anti-tick vaccines containing immune-protective epitopes, should be considered for more effective anti-tick vaccine formulations against a wide range of ticks [17, 65, 69–71].

Importantly, the efficacy of the mono-antigenic and cocktail vaccines in this study was similar to that obtained with Bm86, which achieved > 90% efficacy against several ticks [18]. Cocktail vaccines could serve as a reliable immunization strategy and therefore warrant further studies to evaluate their potential as formulations for anti-tick vaccines under field conditions.

## Conclusions

We demonstrated that immunization with rSUB and rFER2 as mono-antigens as well in a cocktail (rSUB+rFER2+rP0), as vaccines, triggered a significant immune response that affected various biological parameters of *R. sanguineus* s.l. ticks. The results presented here support the development of cross-protective and cocktail-based anti-tick vaccines that have the potential to be valuable tools in the development of a universal anti-tick vaccine. Field trials are necessary to demonstrate the efficacy of these vaccines, which could simultaneously target a broad range of tick species under natural conditions.

### Supplementary Information

**Additional file 1: Figure S1.** SDS-PAGE and western blot analysis of heterologous expression of tick proteins. Lanes M and B indicate the protein marker and bovine serum albumin, respectively.** A** rSUB. Lanes: 1, 2, induced cell lysate; 3, 4, purified rSUB protein.** B** rFER2. Lane: R, purified rFER2 protein.** C** rP0. Lanes: 1, 2, induced cell lysate; 3, 4, rP0 protein; 5–7, Purified rP0 proteins. Arrows indicated the purified recombinant proteins.

**Additional file 2: Table S1.** Sera antibody titers of rabbits immunized with rSUB, rFER2 and cocktail (rSUB, rFER2, rP0) after three doses.

## Data Availability

The datasets supporting the conclusions of this article are included within the article.
