# Supplementary figures and images for: Cross-species immunoprotective antigens (subolesin, ferritin 2 and P0) provide protection against Rhipicephalus sanguineus sensu lato
Source: Parasit Vectors. 2024 Jan 3;17:3. doi: 10.1186/s13071-023-06079-3 (PMC10765945; doi:10.1186/s13071-023-06079-3)

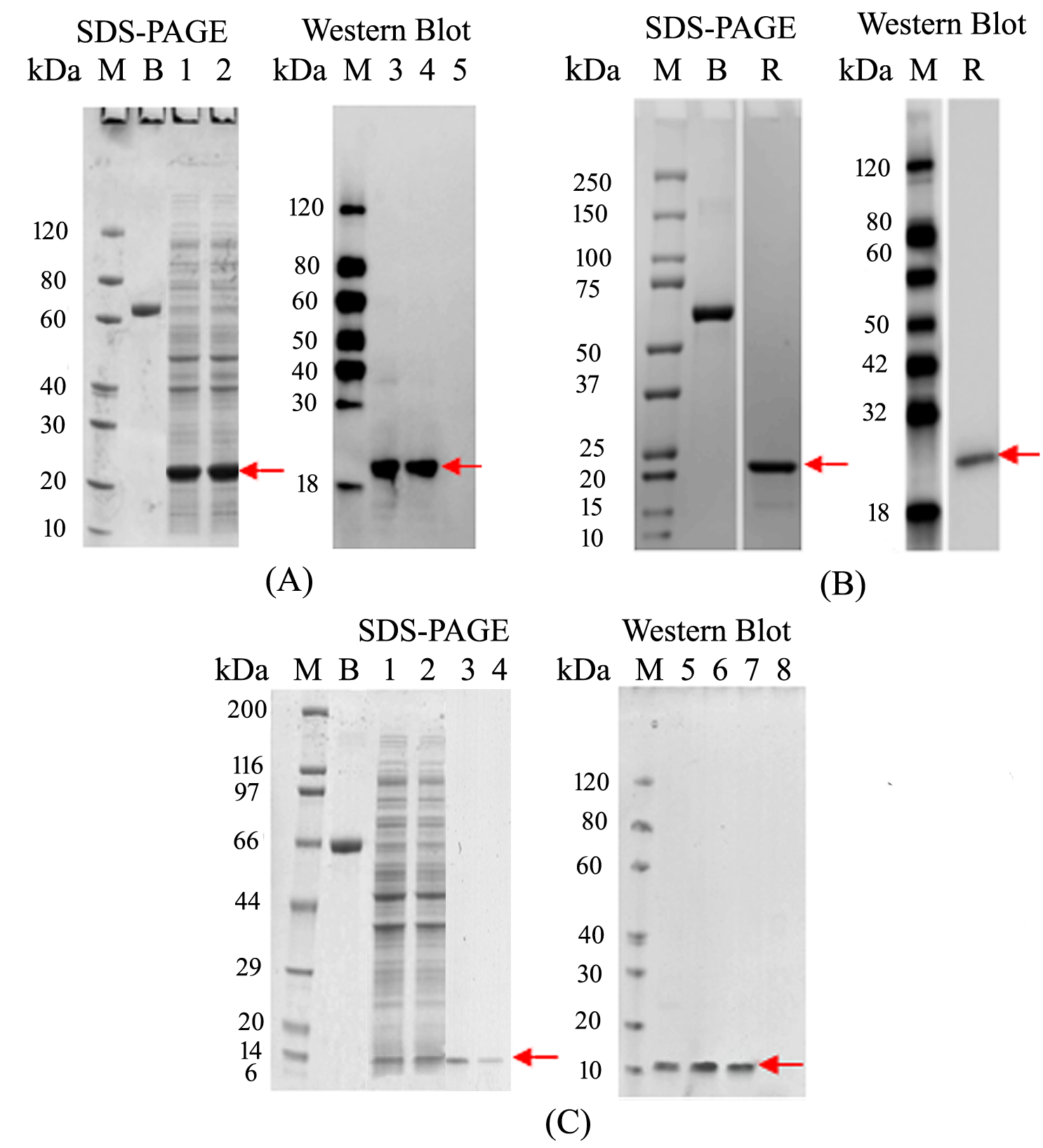

Supplement: Supplementary file 1 — Additional file 1: Figure S1. SDS-PAGE and western blot analysis of heterologous expression of tick proteins. Lanes M and B indicate the protein marker and bovine serum albumin, respectively. A rSUB. Lanes: 1, 2, induced cell lysate; 3, 4, purified rSUB protein. B rFER2. Lane: R, purified rFER2 protein. C rP0. Lanes: 1, 2, induced cell lysate; 3, 4, rP0 protein; 5–7, Purified rP0 proteins. Arrows indicated the purified recombinant proteins. [file 13071_2023_6079_MOESM1_ESM.tif]
