# Supplementary material for: Cross-species immunoprotective antigens (subolesin, ferritin 2 and P0) provide protection against Rhipicephalus sanguineus sensu lato
Source: Parasit Vectors. 2024 Jan 3;17:3. doi: 10.1186/s13071-023-06079-3 (PMC10765945; doi:10.1186/s13071-023-06079-3)
Supplement: Supplementary file 2 — Additional file 2: Table S1. Sera antibody titers of rabbits immunized with rSUB, rFER2 and cocktail (rSUB, rFER2, rP0) after three doses. [file 13071_2023_6079_MOESM2_ESM.docx]

**Table S1.** Sera antibody titers of rabbits immunized with rSUB, rFER2, and cocktail (rSUB, rFER2, rP0) after three doses.

| Proteins | Rabbit/sera antibody titers |
| --- | --- |
| SUB | SUB1/256,000  SUB2/4,096,000  SUB3/4,096,000  COK1/1,024,000  COK2/2,048,000  COK3/2,048,000 |
| FER2 | Fer1/8,192,000  Fer2/8,192,000  Fer3/8,192,000  COK1/8,192,000  COK2/8,192,000  COK3/8,192,000 |
| P0 | P0 1/4,096,000  P0 2/128,000  P0 3/2,048,000 |
